# A Unified Model of Heading and Path Perception in Primate MSTd

**DOI:** 10.1371/journal.pcbi.1003476

**Published:** 2014-02-20

**Authors:** Oliver W. Layton, N. Andrew Browning

**Affiliations:** 1 Center for Computational Neuroscience and Neural Technology, Boston University, Boston, Massachusetts, United States of America; 2 Scientific Systems Company Inc. (SSCI), Woburn, Massachusetts, United Sates of America; Philipps-University Marburg, Germany

## Abstract

Self-motion, steering, and obstacle avoidance during navigation in the real world require humans to travel along curved paths. Many perceptual models have been proposed that focus on heading, which specifies the direction of travel along straight paths, but not on path curvature, which humans accurately perceive and is critical to everyday locomotion. In primates, including humans, dorsal medial superior temporal area (MSTd) has been implicated in heading perception. However, the majority of MSTd neurons respond optimally to spiral patterns, rather than to the radial expansion patterns associated with heading. No existing theory of curved path perception explains the neural mechanisms by which humans accurately assess path and no functional role for spiral-tuned cells has yet been proposed. Here we present a computational model that demonstrates how the continuum of observed cells (radial to circular) in MSTd can simultaneously code curvature and heading across the neural population. Curvature is encoded through the spirality of the most active cell, and heading is encoded through the visuotopic location of the center of the most active cell's receptive field. Model curvature and heading errors fit those made by humans. Our model challenges the view that the function of MSTd is heading estimation, based on our analysis we claim that it is primarily concerned with trajectory estimation and the simultaneous representation of both curvature and heading. In our model, temporal dynamics afford time-history in the neural representation of optic flow, which may modulate its structure. This has far-reaching implications for the interpretation of studies that assume that optic flow is, and should be, represented as an instantaneous vector field. Our results suggest that spiral motion patterns that emerge in spatio-temporal optic flow are essential for guiding self-motion along complex trajectories, and that cells in MSTd are specifically tuned to extract complex trajectory estimation from flow.

## Author Summary

Much human and primate psychological and electrophysiological research on visually-guided navigation has focused on heading perception, defined as the instantaneous direction of travel. However, the perception of path of travel, or trajectory, is arguably more important, because it informs in a more general sense whether the observer is on a collision course with moving objects or will intercept a target. In the present article, we describe a theory based on physiological evidence of how primate visual area MSTd may simultaneously and dynamically encode heading and path. The model connects many different sources of data, including psychophysics on human perception of heading and path with and without eye movements, and primate electrophysiological data on path-selective cells in MSTd. We propose neural mechanisms explaining why humans report traveling along curved paths when the display represents a straight path with simulated eye rotations. We predict that perceptual sensitivity to heading and path emerges in primate MSTd through the dynamical and competitive interactions between neurons tuned to the continuum of spiral-radial patterns.

## Introduction

**Figure 1:**
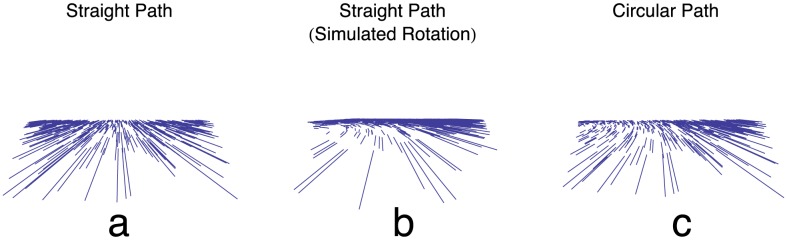
Exemplar first-order optic flow fields. (a) Radially expanding optic flow experienced by an observer traveling along a straight path on a ground plane. The optic flow contains the focus of expansion (FoE) singularity on the horizon, which indicates the heading direction. (b) Movement of an observer along a straight path, as in (a), but with a constant amount of rotation added to the first-order optic flow (simulated rotation condition). Human subjects that view displays with simulated rotation report traveling along a circular path. (c) First-order optic flow experienced by an observer traveling on a circular path whose gaze is along heading or tangent to the circular path.

Gibson noted that animals can navigate about their environment using the changing pattern of light distributions falling on the retina, which is now known as *optic flow* [1]. Travel parallel to a ground surface, along a straight path, without eye movements or body rotations produces characteristic patterns of optic flow, which Gibson called a “melon-shaped family of curves” (Figure 1a). These flow patterns contain a singularity known as the *focus of expansion (FoE)*. When the path of travel is straight, the optic flow field expands radially and the FoE specifies the direction in which the animal is going (heading). Gibson observed that animals could navigate using optic flow by aligning the FoE with the direction in which the animal wishes to travel.

Since Gibson proposed this strategy for navigation, much psychophysical research has focused on understanding human perception of heading [2]. For the remainder of this article, we define *heading* to refer to the instantaneous direction of travel of an observer, and we define *curvilinear path* as the trajectory of travel, which may be curved. Psychophysical studies of human heading judgments have largely been based on static environments, consisting of dot or textured ground planes or 3D dot clouds. The observer typically travels along a straight path. In such environments, humans accurately judge their heading, within 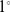 [3]–[5]. Biologically inspired models of human heading perception often make use of depth variations in the visual scene (motion parallax) [6], [7] to estimate the observer's heading given a two dimensional (2D) retinal velocity field. Template matching [8], [9], whereby the retinal optic flow is compared to a number of large-field radial expansion patterns, or a combination of the approaches is also used.

Navigation under natural conditions is more complex than traveling on a straight path without any rotation. When the observer travels along a straight path, factors, such as eye movements and gaze, introduce rotation, which may result in optic flow that is not radially expanding or contracting. Sources of rotation are either considered *retinal* or *extra-retinal* [10]. Rotations that occur through the actions of the observer, such as eye, head, or body movements, are considered extra-retinal, whereas rotations due to path curvature are considered retinal. Research on heading perception during smooth-pursuit eye movements shows that human bias in heading judgments remains constant 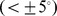 and independent of angular rotation due to eye movement velocities [11], [12]. However, when human subjects fixate (no extra-retinal rotation) and are shown optic flow displays that simulate what would be seen by an observer traveling along a straight path with a constant amount of rotation (Figure 1b), humans make large errors in heading judgments that is proportional to the rate of rotation [11]. This is often referred to as the *simulated rotation condition*, in which the retinal rotation experienced by human observers is due to the simulated eye movements. Subjects typically note the experience of traveling along a curved path and not a straight path with eye rotation (compare Figure 1b and 1c), which is the assumption of the experimenters. The large heading bias in the simulated rotation condition may therefore arise through the reporting of the subject's path rather than heading or ambiguity in the task instructions [13], [14]. Mathematical analyses indicate that the optic flow experienced by subjects when eye movements are simulated is similar to that experienced traveling on a curved path over the time period of a typical experimental trial [15]. For longer viewing times, the optic flow in the two scenarios diverges and could potentially allow the subjects to disambiguate curved paths from simulated eye rotations.

Electrophysiological evidence suggests that neurons sensitive to radial expansion in MSTd, which are thought to encode heading, demonstrate modulation due to extra-retinal eye signals [16]–[18]. The modest human bias demonstrated by humans while performing eye movements is less than would be expected given the magnitude of the rotations. Assuming MSTd is involved in heading perception, this could be explained by the extra-retinal signals in MSTd imperfectly ‘canceling out’ the rotations. Computational models have employed gain fields in MSTd as the mechanism by which this ‘canceling out’ of rotation may occur [19], [20].

Animals navigate over complex terrain and the path of travel is rarely straight [21], [22], but few studies have examined human navigation along *curved paths*. Those that do tend to examine human path perception in the context of circular paths [23]–[28]. In the present article, if the *path* is not straight, we make the assumption of a circular curved path (Figure 1c). During movement along a curved path, heading and path refer to different characteristics of the observer movement. Heading refers to the instantaneous direction of travel in the world reference frame. From the perspective of the observer, the heading corresponds to the straightaway direction if the curved path were abandoned. Along a circular path, the observer heading is always tangent to circle, aligned in the direction of the clockwise (CW) or counter-clockwise (CCW) traversal. Path corresponds to the fixed curvature trajectory traversed by the observer in world coordinates. Both heading and path are independent of the observer gaze and body orientation.

When traveling along a curved path without eye or body movements, all rotation in the retinal optic flow is due to the path curvature. Research indicates that in environments composed of random dots, humans can accurately judge the curvature of their path in static environments [27], [29], [30]. Judgments remain accurate in the presence of independently moving objects [23], when the observer gaze or instantaneous heading direction and body orientation are always tangent to the path of travel. This naturally occurs during locomotion. Human judgments of path curvature are not affected by whether the environment is composed of sparse dots, limited lifetime dots, or dense textures [31]. However, many studies that investigate curvilinear navigation are confounded by whether subjects report heading or future path [14], [32]–[34].

### Theories of Path Perception

Existing theories of path perception are a set of heuristics that do not specify the mechanisms by which the path is perceived in the brain. Some theories depend on the active tracking of ‘features’ in the visual scene [27], [35], while others implicate an extensive cognitive component, such as updating estimates with respect to external reference objects [31], [36].

We first summarize the former class of path perception theories. The *passing flow line* hypothesis observes that optic flow integrated over an extended period of time yields a streamline that passes underneath the observer and coincides with the path of travel [35]. This hypothesis assumes that the observer gaze is in the direction of heading and requires the environment to have a textured ground plane passing directly underneath the observer. A related hypothesis proposed by Wann and Swapp, which we call the *vertical vector* hypothesis, notes that if the observer maintains gaze on the destination of travel, the path can be recovered from retinal flow by integrating first-order flow vectors that are vertically aligned [37]. This strategy does not require knowledge of heading. The *vertical flow line* hypothesis posits that the visual system tracks the constellation of vertical optic flow streamlines that exist when the observer fixates a point on the future path. This strategy assumes that humans fixate on their destination while traveling along curvilinear paths. The *reversal boundary* hypothesis notes that the future path of travel coincides with direction reversals or “zero-crossings” in the horizontal motion component once the optic flow has been projected onto the retina [27]; the horizontal motion component of texture inside (outside) the path will be rightward (leftward), or vice versa depending on whether the circle is traversed CW or CCW. While psychophysical evidence suggests that humans are most accurate in judging path curvature when the gaze direction is aligned with the heading, it is not clear how this strategy could tolerate momentary fluctuations in gaze. Warren and colleagues have proposed a *vector normal* hypothesis whereby the center of the circular path can be determined by computing the intersection of the vector normals of two points in the environment [27]. Using the vector normals, knowledge of the circular path center, and the observer's current position, the radius and therefore the curvature of the path of travel can be recovered.

The hypotheses reviewed above suffer from rigid constraints on the environment or observer gaze, and are unlikely to represent general theories of human path perception. The strategies proposed by the passing flow line, vector normal, and vertical vector hypotheses only hold when observers look where they are going—i.e. gaze is along the heading direction. These hypotheses cannot account for activities that presumably depend on the perception of path, such as steering a vehicle [38], for which successful control of navigation accompanies natural changes in gaze [37]. Humans perceive their path of travel in sparse environments composed of small quantities of dots. The boundary reversal hypothesis, however, requires dense optic flow to ascertain the horizontal motion zero-crossing. From a neural computation point of view, it is unclear how the brain could track the context-specific local features proposed by any of the above hypotheses over time.

The following path estimation theories rely on external landmarks in the environment. The *reference object* hypothesis posits that observers either update their position or integrate the change in heading over time with respect to an object embedded in the environment [36]. Subjects in the experiments of Li and Cheng were able to judge their future path of travel in the absence of persistent objects in the environment, rendering the reference object hypothesis an incomplete strategy [31]. Li and Cheng tested whether humans can integrate the change in heading without a reference object by tracking the “drift” in the FoE over time with gaze remained fixed along a particular axis when the observer travels along the circle without any rotation (Z-axis condition). Subject responses were consistent with the percept of moving along a straight rather than a circular path, making the *FoE drift* hypothesis unlikely [31]. Finally, Li and Cheng proposed that observers first estimate heading to established a reference, then estimate the path curvature, which is mathematically defined for a circular path as the ratio between the rotation and translation rates [31]. It is not clear if, or how, mechanisms in the brain could perform these operations.

In summary, theories of path perception either treat path perception as independent of heading or depend on its prior estimation. In the present article, we propose a neural model of the primate visual system in which representations of heading and path are determined simultaneously and dynamically interact in the same population of neurons.

### Neurophysiology of Path Perception

Neurons in the primate medial superior temporal area (MST) of the superior temporal sulcus (STS) exhibit tuning in the laboratory to radially expanding optic flow patterns, similar to those experienced by an observer moving forward on a straight path. MSTd cells have therefore been the focus of neurophysiological investigations of the mechanisms underlying visually-guided navigation. MST is the earliest visual area, the fewest synapses away from the retina in the primate dorsal stream, that responds to large field pattern motion. Evidence suggests that MST in monkey is composed of functionally distinct dorsal (MSTd) and ventral (MSTv) regions. Whereas neurons in MSTd exhibit sensitivity to optic flow patterns that occupy areas of the visual field as large as 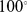, MSTv neurons have smaller receptive field sizes and are suspected to be involved in the perception of object motion [39]. MSTd neurons demonstrate sensitivity to dot speed [40] and spatial shifts in FoE position [41], and therefore are thought to be involved in heading perception [42].

**Figure 2:**
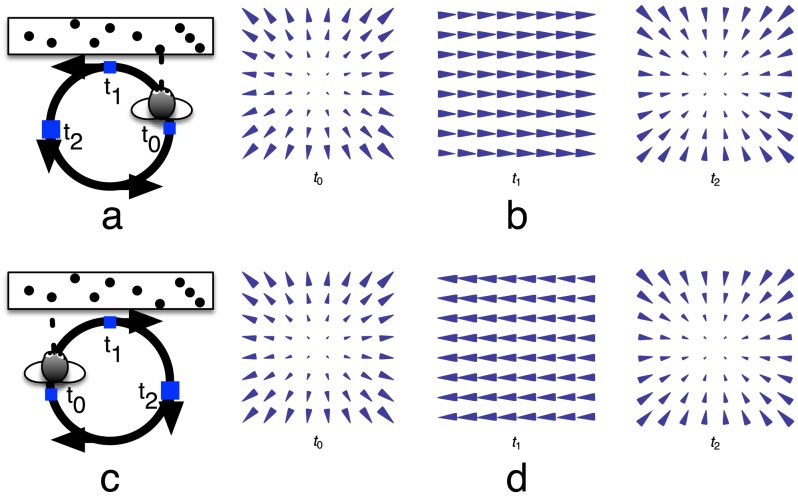
Experimental paradigm and sample optic flow fields from Froehler and Duffy, who report the existence of path selective neurons in MSTd [43]. A monkey seated in a sled traveled CCW (a) or CW (c) along a circular track while maintaining gaze on the distal wall of luminous dots. The body, head, and eye did not rotate so that the monkey always directly faced the distal wall. The monkey therefore experienced radially expanding or contracting optic flow without sources of rotation. (b,d) Instantaneous optic flow experienced by the monkey at different locations along the circular track. In (b) at 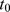, the monkey views a radially expanding optic flow while moving CCW when the heading direction is straight ahead, which is the same as the optic flow viewed CW 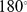 on the other side of the circle. Between 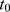 and 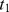, the FoE drifts rightward until at 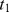 it is out of view. At 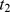, the monkey experiences radial contraction.

Froehler and Duffy have conducted the only neurophysiological study to date that reports the existence of “path selective” neurons in cortex [43]. Monkeys were placed on a sled in a dark room that contained bright dots on the three walls that were within view. The sled moved CW or CCW along a circular path (Figure 2). The sled was configured not to rotate the body as it traversed the circular path. The monkeys maintained gaze, throughout the trial, on a target that was projected from the sled onto the distal wall. Because the projector was attached to the sled and the monkey was trained to maintain gaze on the target, the fixation point occupied the same position within the monkeys' visual field over time. The optic flow experienced by the monkeys contained no rotation and appeared to radially expand or contract at each instant, with a FoE or focus of contraction (FoC) that ‘drifted’ horizontally during the trial. A monkey traveling once around the circle on the sled therefore viewed a sequence of headings and each had an equivalent at antipodal positions in both the CW and CCW trials. Froehler and Duffy recorded from single neurons in MSTd and 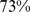 elicited differential activity at antipodal positions on the track, where expansion/contraction optic flow patterns were identical. The neurons' response depended on whether the circle was traversed CW or CCW, and as a result the authors claimed these cells demonstrated path selectivity. The authors also found heading selective cells, which fired when the optic flow contained their preferred heading irrespective of the CW or CCW traversal direction, and place selective cells, which responded when the monkey moved to a particular location of the room irrespective of the visual motion pattern. The selectivity of neurons in the sample was distributed along a continuum, ranging from those demonstrating high (path selective) to those demonstrating low (heading selective) CW v.s. CCW differential activity. The mechanisms that underlie how these cells in MSTd respond along a continuum to heading and path were not evaluated by the study.

In summary, neurons in MSTd demonstrate tuning to optic patterns, similar to those that would be viewed by an observer traveling on a straight path, and may exhibit sensitivity to path in the absence of rotation [43]. Locomotion along curved paths typically involves rotation, so, if the neurons discovered by Froehler and Duffy are in fact path-selective, it remains unclear how their response patterns would generalize to more natural movement conditions. Our model proposes mechanisms by which the MSTd neurons identified by Froehler and Duffy elicit differential firing rates when the instantaneous visual motion appears the same, yet the monkey moves CW or CCW around the circle. Our analysis integrates the findings with other known properties of MSTd neurons.

### Spiral-Selective MSTd Cells Dynamically Encode Path and Heading Direction

**Figure 3:**
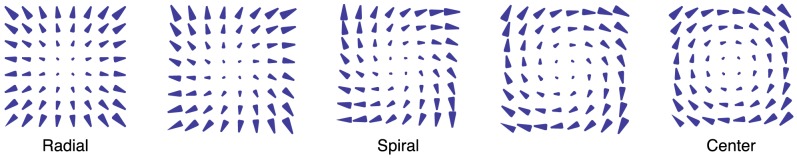
Spiral space continuum of motion patterns employed in electrophysiological studies to probe cell selectivity to spiral motion. Sensitivity to radial expansion and center motion is tested by the left and right ends of the continuum, respectively. Spiral patterns exist in between as an interpolation between the radial and center patterns. The spiral space also contains contracting spirals and those with CCW orientations (not shown).

If the primary role of MSTd were to determine heading, most MSTd neurons would be expected to preferentially respond to radial expansion and contraction. While many neurons in MSTd are tuned to such patterns, many others exhibit preferential responses to patterns in a spiral space spanned by radial and center templates (Figure 3). Moreover, neurons in MSTd would be expected to discount retinal rotation, as many appear to do with extra-retinal rotation [16]. However, Orban and colleagues demonstrated that MSTd neurons tuned to radial expansion did not respond to expansion displays when adding simulated retinal rotation [44]. Therefore, rotation does not appear to be discounted in MSTd neurons when the source is retinal rather than extra-retinal. Graziano and colleagues found that more neurons preferentially responded to CW and CCW spirals than to rotation or contraction, and the tuning curve width and selectivity did not differ across the MSTd population for radial, spiral, and center patterns [45]. That is, neurons tuned to radial expansion did not exhibit sharper tuning curves than those tuned to spirals. Spiral tuning also appears in neurons in the ventral parietal area (VIP) [46] and area 7a [47], two of the brain regions to which MSTd projects [48]. Despite the diversity of tuning in MSTd, no well-defined hypothesis has been proposed for the functional role of MSTd neurons tuned to spiral patterns. Graziano and colleagues speculated that spiral tuning may allow MSTd to detect a rotating moving object or perceive the pattern of motion experienced by walking forward while tracking a point on the ground, however, these hypotheses are unproven.

We claim that selectivity to optic flow across a spiral space continuum simultaneously affords MSTd with sensitivity to the curvature of the path and to the heading direction. When an observer travels along a curvilinear path on a ground plane with a fixed direction of gaze, a spiral-like pattern is experienced and optic flow contains rotation that specifies the path curvature [31]. Theoretically, spiral selective neurons should be sensitive to the curvature of their preferred spiral pattern and would therefore be capable of extracting information about the future path. Although the actual representation in the brain is unlikely to resemble an abstractly-defined mathematical spiral space [49], we assume that the spiral space selectivity in MSTd spans the continuum between radial and center patterns that has been electrophysiologically tested in numerous studies [44]–[47]. Because we developed a neurophysiological model, it is important to constrain the model to constructs that can be verified by data. Since no neurophysiological data exists with more ecological stimuli we would be unable to verify a model design constructed using such templates designed to reflect our intuitive understanding of the ecologically valid space.

**Figure 4:**
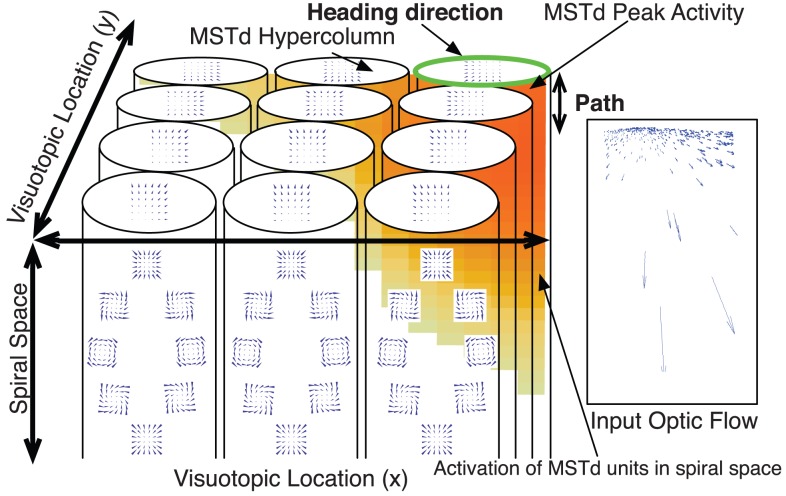
Schematic depiction of the model coding of path curvature and heading in MSTd. Neurons in a model MSTd hypercolumn possess selectivities across a spiral space spanning CW, CCW, radial expansion, radial contraction, and center motion patterns. The length and width dimensions of the schematic MSTd selectivity volume correspond to neurons with 2D visuotopic tuning. Therefore, at every position in the visual field, there is a model MSTd hypercolumn with a full set of units tuned to radial, spiral, and center optic flow patterns. For example, the hypercolumn on the top left corresponds to MSTd units with receptive fields centered on the top left portion of the visual field, which have focus of expansion or center of motion tuning in that location. Travel along a circular path elicits a distribution of activity within the MSTd volume (overlaid heat map). The position of the activity peak across the volume in the spiral space (depth) dimension corresponds to the model path curvature estimate, and the 2D position of the peak in the spatial dimensions (length and width) indicates the estimated heading direction.

Figure 4 shows a visualization of the proposed functional organization of MSTd. Each cylindrical volume represents a functional MSTd hypercolumn with respect to spiral selectivity. A hypercolumn contains a subpopulation of MSTd neurons that are sensitive to a spectrum of optic flow patterns in spiral space that have receptive fields centered at the same location of visuotopic space. The horizontal (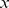) and vertical (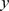) axes specify the spatial dimensions of the MSTd visuotopic map. Each point in this two-dimensional space indicates tuning to a FoE, FoC, or more generally a center of motion (CoM) in that particular visuotopic location—irrespective of the pattern selectivity. For example, the top-right hypercolumn in Figure 4 contains subpopulations of MSTd neurons tuned to motion patterns (e.g. radial expansion, radial contraction, spiral, center) that have the CoM located on the top-right region of the visual field. The axis than spans the depth of the hypercolumn represents the degree of spiral tuning for the subpopulation of neurons that have receptive fields centered at a particular location of the visual field. MSTd neurons may exhibit tuning to CW or CCW spiral patterns that either expand or contract. Spiral patterns smoothly vary in ‘spirality’ between patterns that are radial with no curvature (top and bottom), and those that are centers (left and right). We propose that the ‘spirality’ of the most active subpopulation of neurons in MSTd encodes the curvature of the path, and the two-dimensional visuotopic position of that maximally active subpopulation represents the heading.

In the simple case of travel along a straight path, we expect neurons tuned to radial expansion to be most active, indicating no path curvature, and we anticipate the peak to spatially coincide with the FoE to indicate the heading. Therefore, the population MSTd response in this example is the same as if there were only neurons selective to radial patterns. In the case of a circular path, we expect the spiral-selective neurons with spiral arms that best match the path curvature to be most active. As reported in several psychophysical studies [11], [31], different gaze patterns modulate the rotation present in the optic flow. In the present paper, we test whether the maximal activity of neurons tuned in spiral space maps onto human judgments of path curvature as gaze varies.

We present a dynamical model of primate MSTd that builds on electrophysiological findings and explains a range of human psychophysical data on path and heading perception with and without eye movements. The main goal is to present a mechanistic hypothesis of path perception that provides a unified framework to interpret psychophysical and neurophysiological data on heading and path perception. Our model goes beyond existing heuristics by providing a mathematical description and biologically-plausible implementation that is readily testable. Our analysis and simulations show that the model yields performance similar to humans under different gaze conditions, circular path radii, and eye movement patterns. The model predicts that the neurons reported by Froehler and Duffy obtain their path selectivity through spiral pattern tuning [43].

## Materials and Methods

Our objective was to create a biologically plausible model of the primate visual system that demonstrates the mechanisms by which perception of heading and path may arise from populations and systems of neurons that process optic flow. The model consists of systems of *shunting* differential equations, each of which models the activity neurons in cortex [50]. This architecture affords realistic neural temporal and competitive dynamics, including recurrent competition and feedback, gain control, and normalization. By creating a computational model using known functional properties of neurons in the magnocellular pathway of the dorsal stream, we can simultaneously connect neurophysiological mechanisms to human data and our test our hypothesis on diverse types of psychophysical data.

### Model Area Descriptions

**Figure 5:**
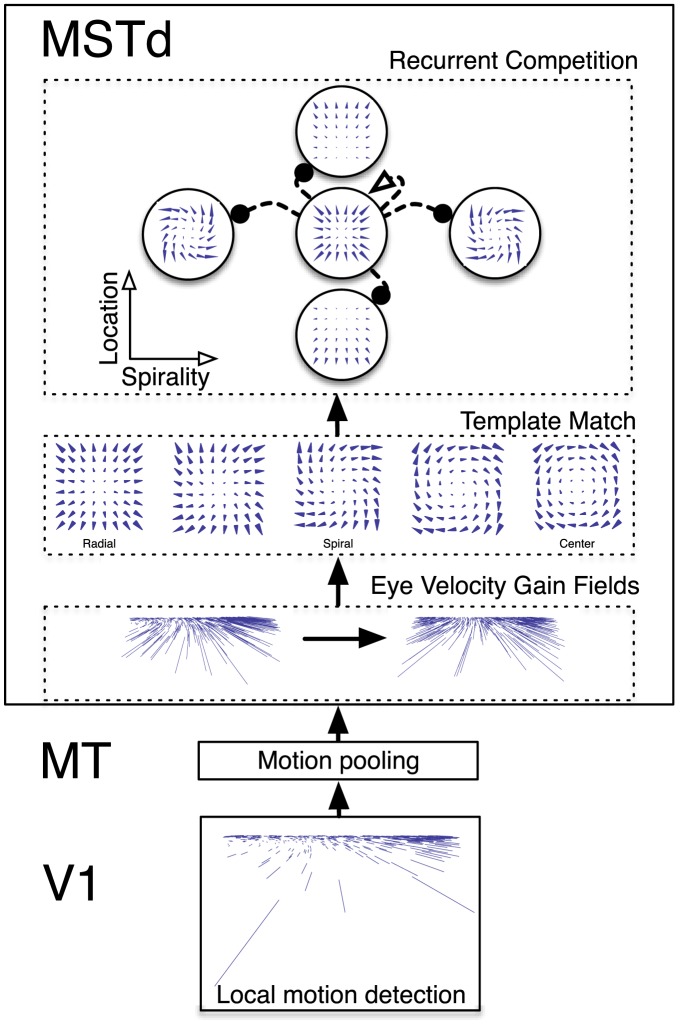
Diagram of model V1-MT-MSTd. First-order local motion is computed in model V1. Model MT receives projections and spatially pools motion signals from model V1. A extra-retinal eye velocity gain field acts on the afferent signals from model MT in MSTd, which compensates for rotation introduced by pursuit eye movements proportional to the eye movement speed in the direction opposite that of the eye movement. A template match occurs in model MSTd, whereby the similarity is assessed between the afferent motion signal and motion field templates sampled in spiral space. A distance-dependent weighting exponentially discounts vector matches by distance from the template singularity. Finally, neurons selective to different spiral patterns, expansion and contraction, CW and CCW orientations, and 2D visuotopic location compete.

The proposed neural model contains three stages that correspond to primate primary visual cortex (V1), medial temporal area (MT), and the dorsal medial superior temporal area (MSTd) (Figure 5). In this paper, we do not model retinal input, but rather use analytical equations to model the vector-based optic flow representation in V1 [51]. A prior version of the model demonstrates how retinal inputs are processed through neural circuits to generate those representations [52], [53]

#### V1 (Local motion detection)

We generated videos of dots distributed on a ground or frontoparallel plane, which served as input to the model. The videos approximate the visual displays shown to human subjects in psychophysical experiments that assess human heading and path perception. The local motion of the dots was computed according to a planar pin-hole camera model [54]. The following equation describes first-order optic flow with translation vector 
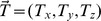
 and rotation vector 
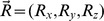
 [55]:
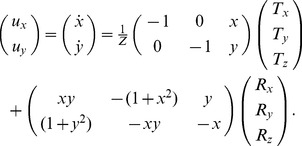
(1)The representation in model V1 computed by Eq. 1 describes the instantaneous velocity 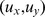 of each projected dot. In Eq. 1, 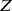 signifies the depth of the projected dot in the world and 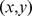 correspond to the spatial position in the 2D projection plane. Values for the parameters 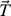 and 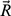 varied according to psychophysical conditions, as described in Experimental Descriptions. For simplicity we use a Cartesian representation of space in V1, although prior work has demonstrated how motion can be processed with cortical magnification [20].

#### MT (Motion pooling)

Model MT units pool the V1 response vectors 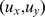 component-wise with a Gaussian receptive field kernel 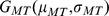. We configured model MT neurons with 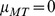, 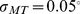, and radius 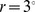, as in previous work to mimic the larger receptive fields in MT compared to V1 [51]. Model MT units respond to large fields of uniform motion and project to MSTd. The pooled model V1 activity in model MT is denoted 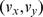.

#### MSTd (Gain fields, spiral template matching, recurrent competition)

Model MSTd consists of three stages: 1) eye velocity gain fields, 2) template matching in spiral space, and 3) dynamical recurrent competition. When the eye velocity is nonzero (e.g. during a smooth-pursuit eye movement), the extra-retinal signal 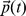 acts presynaptically to MSTd [19], [20]:
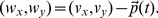
(2)In Eq. 2, 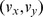 represents the output of model MT and 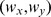 describes the signal after extra-retinal modulation. We simulated the conditions of Cheng and Li, whereby subjects made judgments about their future curvilinear path while visually tracking a horizontally moving target [25]. Because the target moved at a constant velocity and the experimenters discarded data 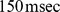 from the onset of the eye movement, we set 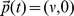, where 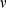 is proportional to the mean pursuit eye movement speed across subjects in each respective condition. The sign depends on the eye movement direction.

We generated spiral templates that spanned the entire visual field by interpolating radial and center vector field patterns (Figure 3) [56]. Eq. 3 defines a radial field 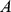 and a center field 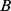:
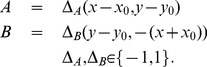
(3)Radial expansion and contraction templates are obtained by setting 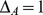 and 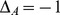, respectively. Center templates with CW and CCW orientations are constructed by setting 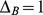 and 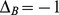, respectively. The values of 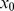 and 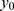 determine the horizontal and vertical spatial offset of the FoE in the radial template and the center of motion (CoM) in the center field. Eq. 4 defines a spiral template, and the value of 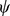 determines the degree of *spirality*, with 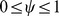.
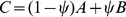
(4)When 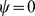, the template is radial, when 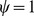, the template is a CW center, and 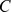 is a spiral template for other values of 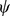.

We created a neural model with 11500 MSTd neurons with motion pattern selectivities determined by the templates in spiral space. The templates were uniformly sampled across the spiral continuum within the visual field 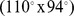. We configured the spatial offsets 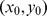 to encompass possible CoM that appeared outside the retinal projection plane in the experiments of Li and Cheng. We found that simulating templates with CoM selectivities far outside the field of view did not impact model performance. This occurred because laminar flow vectors from the template, which are uniform in direction, appear within the field of view and yield poor matches to the optic flow signal. For example, a CCW center template with a CoM selectivity far to the left only responds to uniform vertical motion within the field of view, which does not closely resemble the optic flow experienced while traveling along a curved path. As described below, a model MSTd neuron with weak input from the template matching stage will be suppressed in the competitive dynamics and not affect heading or path curvature estimates produced by the model.

Each model neuron receives afferent signals from model MT, which passes through a template matching stage to assess the degree of similarity between the input signal and model neuron's pattern tuning. The match score at time 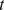, 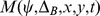, for the neuron at location 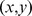 with preferred spirality 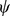 and orientation 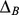 is computed according to the following inner product:
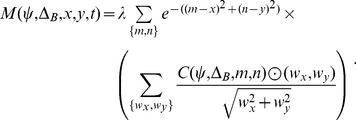
(5)
Eq. 5 computes an inner product (i.e. cosine similarity) by performing component-wise multiplication, indicated by 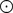, between the input optic flow 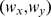 and the spiral template 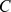. The result is normalized by the 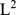 norm of the optic flow vector and the vector components are summed. An exponential distance-dependent weighting is applied to give matches near the CoM greater weight and to afford sensitivity to the CoM position within the visual field, which is consistent with known properties of MSTd neurons [41]. This is followed by a summing over all spatial locations to obtain a scalar match score. Exponential distance dependent weighting has been used in a previous version of the model to balance foveal and peripheral motion components in the grouping of time to contact within an object or surface [57]. The parameter 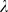 is set to the reciprocal of the number of dots such that the match score is not biased by the number of vector samples.

Eq. 6 defines a dynamical competitive network that describes the activation of model MSTd neuron 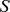 at spatial location 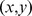 that is selective to a spiral pattern with spirality 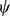 and spiral orientation 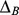 (CW v.s. CCW):
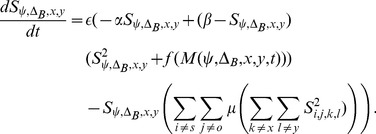
(6)
Eq. 6 is a recurrent competitive field and is configured as a contrast-enhancing or winner-take-all network [50]. Competition between neurons in the network occurs across location and spiral template space. The constant 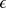 is defined as the reciprocal of the membrane time constant of the model neuron and scales how fast the neuron responds, 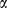 signifies the passive decay rate, and 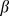 is the saturation upper bound of the model neuron. In Eq. 6, the inhibition model neurons receive from others in the network that have different spiral pattern and orientation sensitivities is set to unity weight, and 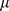 differentially weights the spatial competition. Table 1 summarizes parameters values that were used in configuring the MSTd dynamics. The function 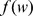 in Eq. 6 is a sigmoidal transfer function defined as
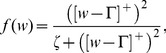
(7)where 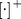 indicates the half-wave rectification 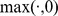, 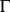 is a threshold on the input from model MT, and 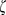 is a sigmoid shape parameter defining the inflection point.

**Table 1:** Parameter values used in simulations.

Parameter	Value	Description
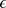	1.0	Inverse cell time constant
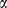	3.25	Passive decay rate
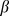	1.0	Activation upper bound
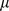	2.5	Strength of inhibition from spatial competition
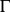	0.01	MSTd presynaptic threshold
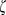	0.07	Sigmoid shape parameter

Path curvature 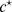 and heading 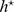 are computed according to Eqs. 8 and 9, respectively, by considering the spirality and spatial position that elicited the maximal MSTd subpopulation activation.
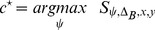
(8)

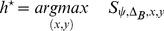
(9)


Note that the argmax operations defined in Eqs. 8–9 are not part of the model computations, and simply allow us to compare the model population activity with judgments made by humans in psychophysical experiments. The MSTd distribution is itself a representation of the confidence that the observer has in the heading direction and path percept. In some senses the distribution itself can be considered a probabalistic read-out, however human subjects were not asked to provide a distribution of confidence over the space so we have no way to validate whether or not our distribution matches human performance. Forcing a decision and taking the maximum likelihood allows us to compare model output against the same forced choice task in humans. We do not claim that the brain decodes information about heading and path distributed across the population in MSTd using maximum likelihood.

Our model does not require the CoM to appear within the field of view to compute heading or path curvature. Model MSTd neurons that possess CoM selectivities outside the field of view estimate heading and path curvature using the available visual information.

All simulations were run on a 8-core 2.66 Ghz Mac Pro with 64 GB of memory using Mathematica 8. Routines involving numerical integration of network dynamics (Eq. 6) and template matching (Eq. 5) were written in C++. Parameter values listed in the text specify those that remained constant throughout all simulations.

### Experimental Descriptions

Unless otherwise noted, all simulation parameters matched those used in the following psychophysical experiment descriptions.

#### Path Perception & Gaze

**Figure 6:**
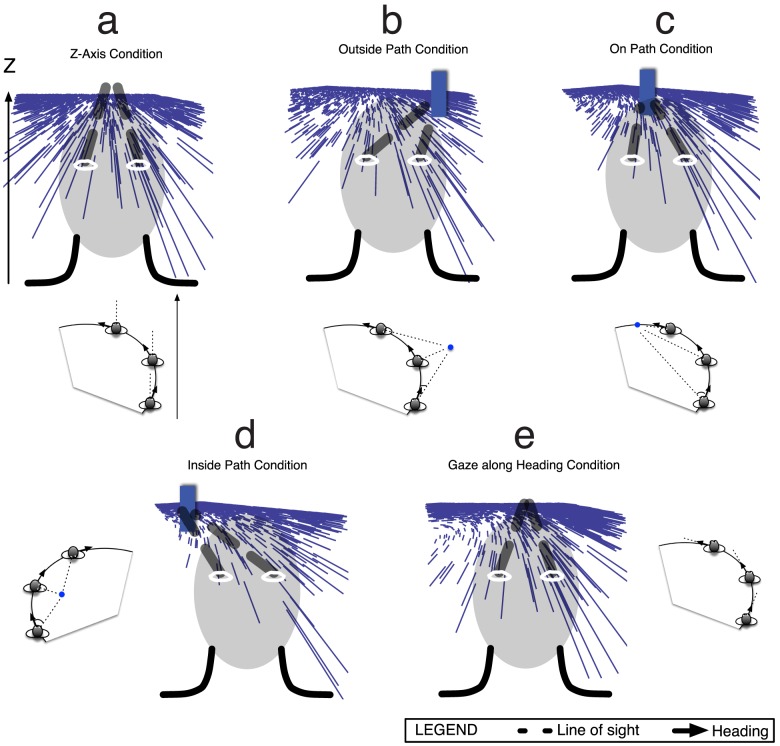
Observer gaze conditions during travel along a circular path tested in the model from Li and Cheng [31]. The gaze in each condition is “simulated” within the computer display because human subjects in the experiments of Li and Cheng maintained fixation throughout the trial. We also tested the model on analogous conditions with pursuit eye movements (see Figure 8). (a) *Z-axis condition*. The observer maintains a fixed body, head, and eye orientation, in the direction of the ‘Z axis’, during travel along the circular path. The optic flow field at every instant is radially expansive, and over time the FoE drifts horizontally. (b) *Outside path condition*. Observer gaze was maintained on a target positioned 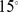 outside the path. (c) *On path condition*. The observer maintained gaze on a target on the future path positioned 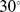 from the initial heading. (d) *Inside path condition*. Observer gaze was maintained on a target positioned 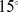 inside the path. (e) *Gaze along heading condition*. Observer gaze is always tangent to the circular path, which is most often the case during human locomotion. Human subjects in the experiments of Li and Cheng underestimated path curvature in the Z axis, outside path, and on path conditions, overestimated path curvature in the inside path condition, and yielded low error in their judgments in the gaze along heading condition.

We simulated the five experimental conditions of Li and Cheng to compare path estimates produced by the model to those produced by human subjects (Figure 6). In the experiment, subjects viewed computer displays that simulated observer travel along a circular path [31]. All coordinates are given with respect to a three-dimensional world coordinate system whereby the origin corresponds to the center of the circular path, the observer begins movement at 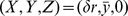, and the observer's position at time 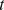 is given by 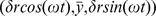, where 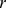 represents the radius of the circular path, the observer either moves CW or CCW about the path, 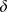 is 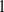 for CCW path traversals and 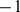 for CW traversals, 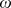 signifies the rate of traversal around the circle, and 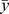 corresponds to the observer eye height. The observer translation vector 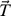 is given by:
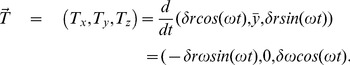
(10)Each trial lasted 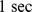 during which time the observer traveled 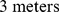 around the circular path. Therefore, we fix 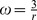. No trial resulted in a traversal greater than a quarter circle. Since the observer motion remained parallel with respect to the XZ plane throughout each trial, 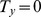. In the experiments of Li and Cheng, the gaze conditions were simulated in the computer display while subjects fixated a stationary fixation cross above the ground plane horizon throughout the trial. Rotation in the optic flow experienced by subjects therefore results from two sources: the curvature of the circular path, and the simulated gaze direction. For example, when a subject fixates a simulated target, rotation occurs due to a combination of gaze and the path curvature. Gaze was simulated to only vary at eye height. Therefore, 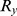 depended on the gaze condition and 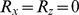.

Each condition was identical except for the simulated observer gaze (i.e. no eye movements). In the *Z-axis condition*, an observer was simulated to travel on a circular path and gaze remained parallel to the Z-axis (Figure 6*a*). The instantaneous vector field contained no rotation, the field at any time appeared to radially expand, and over time the FoE laterally ‘drifted’. Since there was no rotation in the Z-axis condition, 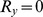. In the *outside path condition*, the simulated gaze was on a target 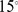 outside the circular path (Figure 6*b*). In this case,
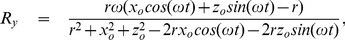
(11)where 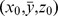 is the position of the target, which was 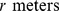 from the observer's initial position (see [58] for derivations). In the *on path condition*, the simulated gaze was on a target 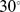 away from the initial heading and 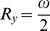 (Figure 6*c*). In the *inside path condition*, the simulated gaze was on a target located 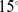 inside the path and 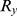 is equal in magnitude but not direction to the value in the outside path condition (Figure 6*d*). The *gaze along heading condition* is the natural case whereby the observer's gaze was simulated to be aligned and rotate with the body and the observer's heading was always tangent to the path, so 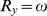 (Figure 6*e*).

For all path conditions, the observer traveled along circular paths with radii 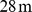, 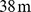, and 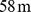. The environment consisted of 200 dots randomly distributed along a ground plane 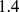
–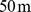 in depth. An analysis of model performance as a function of dot count is shown in Results. We clipped dots that exited the 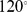 field of view. The computer projector had a 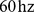 refresh rate, so we simulated observer motion across 60 frames of video. At the end of each trial, subjects manipulated a probe a fixed depth away such to intercept the future path, were the trial to continue. Path error was determined by computing the angular difference between the subject response and future path.

To compare model path estimation performance and human psychophysical data, we must map characteristics from the neural population response in MSTd to human perceptual space. The abscissa of the peak along the spiral pattern continuum (Eq. 8), defines a read-out of the model's representation of path curvature. To compute path error in the model, we subtract model path estimates (Eq. 8) in each gaze condition with that yielded in the gaze along heading condition. Comparing path estimates to that obtained in the gaze along heading condition calibrates the model to the situation wherein judgments of path curvature are accurate. This occurs when humans look where they are going, which is often the case during normal locomotion [26]. No additional transformation, other than the subtraction, was necessary to fit the psychophysical data.

#### Path Perception & Eye Movements

The experiment of Cheng and Li followed the same paradigm as the preceding study [31], but introduced real eye rotations through two conditions in which subjects performed smooth-pursuit eye movements to track a horizontally moving target on the computer display [25]. The *orientation along heading* condition was the same as the gaze along heading condition, except subjects tracked a target moving toward the exterior of the path, which had the effect of linearizing the optic flow [59]. The *orientation along Z-axis* condition was the same as the Z-axis condition, except subjects tracked a target moving toward the interior of the path, which had the effect of adding extra-retinal rotation. The two conditions were configured such that the first-order retinal optic flow appeared the same. In the orientation along heading condition, the mean subject pursuit eye movement speeds were 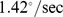, 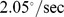, and 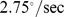 for path rotation rates of 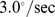, 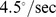, and 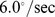. In the orientation along Z-axis condition, the mean subject pursuit eye movement speeds were 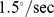, 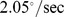, and 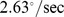 for path rotation rates of 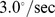, 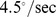, and 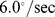.

## Results

### Path Perception and Gaze

**Figure 7:**
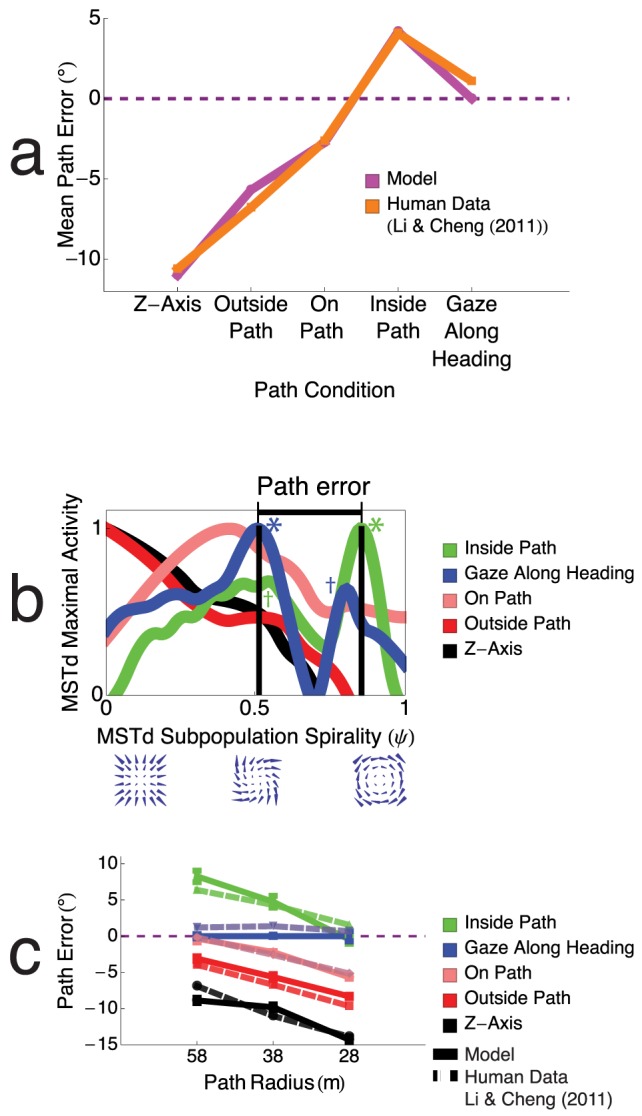
Path errors obtained by the model in the five gaze conditions. (a) Path error averaged across circular path radius. Positive and negative path errors indicate overestimations and underestimations of path curvature, respectively, and zero path error signifies veridical performance. Both humans and the model underestimated path curvature in the Z axis, outside path, and on path conditions, overestimated path curvature in the inside path condition, and elicited near veridical performance in the gaze along heading condition. (b) Model MSTd activity across spiral pattern selectivity space during an exemplar trial with a 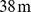 path radius for the five gaze conditions. The location of each peak across the spiral continuum determines the model estimate of path curvature. For example, in the Z-axis condition (black), the MSTd activity peak occurs in the subpopulation sensitive to radial expansion (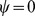), and therefore the model indicates zero path curvature (straight path). (c) Model path errors (solid lines) compared to human data from Li and Cheng (replotted, dashed lines) in the five gaze conditions as a function of path curvature [31]. Model path errors were in good agreement in all gaze conditions with those based on human judgments (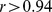), and path error decreased linearly (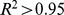) with path curvature. Error bars correspond to standard error of the mean (SEM).

Figure 7a depicts the path error obtained in each experimental condition, averaged across the three path curvatures. The random dot displays in model simulations and the human experiments contained 200 dots.

In the *Z-axis condition*, an observer was simulated to travel on a circular path and gaze remained parallel to the Z-axis (Figure 6*a*). The instantaneous vector field contained no rotation, the field at any time appeared to radially expand, and over time the FoE laterally ‘drifted’. In the *outside path condition*, the simulated gaze was on a target 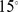 outside the circular path (Figure 6*b*). In the *on path condition*, the simulated gaze was on a target 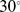 away from the initial heading (Figure 6*c*). In the *inside path condition*, the simulated gaze was on a target located 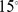 inside the path (Figure 6*d*). The *gaze along heading condition* is the natural case whereby the observer's gaze was simulated to be aligned and rotate with the body and the observer's heading was always tangent to the path (Figure 6*e*).

Positive and negative path errors correspond to overestimations and underestimations of the path curvature, respectively. Zero path error signifies an accurate assessment of path curvature. Mean model path errors agree well with those produced by humans subjects in the experiments of Li and Cheng [31]. The model and human subjects on average underestimated the path curvature in the Z-axis, outside path, and on path conditions, overestimated the path curvature in the inside path condition, and accurately judged the path curvature when gaze was aligned with the heading direction.

When optic flow experienced by an observer moving along a curvilinear path is presented to the model, a subpopulation of units in a particular model MSTd hypercolumn becomes most active (Figure 4). Path curvature is coded by the spiral tuning of these most active units in MSTd. The visuotopic tuning of this maximally active subpopulation does not impact the encoding of path curvature. Figure 7b plots the peak magnitude of each MSTd unit tuned to a different template in spiral space, irrespective of the unit's tuning in visuotopic space, in the five gaze conditions when the path curvature was 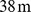. The 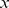 axis corresponds to the pattern tuning across the spiral space continuum, and the 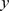 axis shows the maximal activity elicited by units sensitive to a particular optic flow pattern in spiral space, irrespective of its visuotopic tuning. A spirality of 0 signifies a MSTd neuron that is preferentially tuned to radial expansion, a spirality of 1 indicates a tuning to CCW center motion patterns, and intermediate values correspond to preferential responses to CCW spiral patterns. In the *Z-axis* and *outside path* conditions, the maximally active MSTd unit was the one that was sensitive to radial expansion (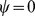). The positions of MSTd activity peaks in the *Z-axis* (black) and *outside path* (red) conditions were to the far left of the spiral space continuum. Radially expansion patterns contain no curvature, therefore, the model signals, similar to humans, in the *Z-axis* and *outside path* conditions that the path is straight.

To compute path error from representations of path curvature in the model, we have to ground the spiral continuum into perceptual space. When humans look where they are going, judgments of path curvature are accurate. This is most often the case during normal locomotion [26], so we calibrate the model around the distribution of activity in model MSTd yielded in the natural gaze along heading condition (Figure 7b, blue). We subtracted the spirality of the peak obtained in each condition (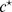) from that obtained in the gaze along heading condition to yield the model path error. We were able to configure the model such that no transformation of the subtraction in spiral space was required to yield the results shown in Figure 7.

The ordinal positions of peaks shown in Figure 7b correspond to path errors made by humans in the experiments of Li and Cheng [31]. As mentioned above, the MSTd activity peaks in the *Z-axis* and *outside path* conditions are produced by units tuned to radial expansion. These peaks are positioned far to the left compared to the activity peak in the *gaze along heading* condition, indicated by the blue *. Subtraction of the abscissae of the peaks yields large magnitude negative path errors, consistent with the large underestimations of path curvature made by human subjects. The position of the activity peak in the *on path* condition (pink) is closer to that in the *gaze along heading* condition (blue *). This yields a negative path error, albeit lower in magnitude than those produced in the *Z-axis* and *outside path* conditions. Therefore, the model signals an underestimation of path curvature, consistent with the judgments of human subjects.

The bimodality observed in the MSTd activity distributions shown in Figure 7b arise due to an interaction between the input optic flow, temporal dynamics, and competition in the model. Retinal flow that contains a large amount of rotation (e.g. inside path condition) yields activity peaks in units tuned to spiralities around 1. Conversely, flow that contains a small amount of rotation (e.g. Z-axis condition) yields activity peaks in units tuned to low spiralities around 0. Due to observer gaze, the rotational component in the optic flow changes over time during travel along the circular path. As a result, the activity peaks in model spiral space, such as those shown in Figure 7b, “move” over time. Subpeaks arise, such as the “ripples” in the green curve, because at one point in the time history, a unit with the corresponding spirality of the subpeaks was most active. Competitive dynamics in the network suppress subpeaks over time. Although peaks in spiral space stabilized in the network, subpeaks were not always completely suppressed by the end of the trial. Because the projected CoM location and rotation in the optic flow vary nonlinearly with time (e.g. Eq. 11), peaks are not always displaced to continuous locations in spiral space. This yields a bimodal distribution. Note that the “valley” between the peaks in the inside path and gaze along heading conditions arose due to the spatio-temporal characteristics of the input optic flow and the MSTd network. Peaks emerged within this region of spiral space when simulating travel along paths with different radii.

Figure 7c compares the average path errors produced by the model (solid lines) with those yielded by human subjects (dashed lines) in the five gaze conditions of Li and Cheng [31]. Model path error is assessed on 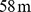, 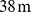, and 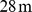 radii circular paths with curvatures of 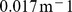, 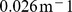, and 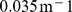, respectively. Error bars in Figure 7c correspond to the standard error of the mean (SEM) yielded over 100 simulations of the model. Our model is deterministic, but the random dot positions in the input introduced variance into the results. Model path estimates produced a good fit to those yielded by human subjects in the Z-axis (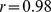), outside path (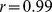), on path (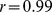), inside path (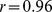), and gaze along heading (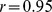) conditions. Similar to human subjects, the model overestimated path curvature when gaze was inside of the path (green) that had the least curvature (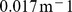). As the path curvature increased, path curvature estimates in the model converged to those obtained in the gaze along heading condition. In the highest path curvature condition, the model path curvature estimates followed the tendency for humans to largely underestimate the path curvature in the on path, outside path, and Z-axis conditions. Across all conditions, the decrease in path error varied as a linear function of increasing path radius (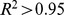).

The dynamics in model MSTd explain why humans overestimate path curvature in the gaze inside path condition along paths with larger radii, but yield more accurate estimates when the path radius is small. In the gaze inside path condition (green), a bimodal distribution emerged in model MSTd. The activity peak occurred to the CCW center side of the spectrum, indicated by the green *, and a subpeak occurred closer to the middle of the spiral continuum, indicated by the green 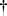 (Figure 7b). Recall that path error is computed in the model by considering the distance between the peaks obtained in a particular condition and in the gaze along heading condition, indicated by the blue *. As shown in Figure 7b, a subpeak exists in the gaze along heading condition, indicated by the blue 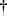, which is close to the peak in the gaze inside path condition (green *). When path curvature increases, more rotation is introduced into the optic flow, which changes the distribution of activity in MSTd spiral space. The subpeak in the gaze along heading condition (blue 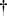), becomes dominant and its proximity to the peak in the gaze inside path condition (green *) results in small path errors. Therefore, high path rotation brings the MSTd peaks (blue 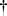 and green *) closer together in the gaze inside path and gaze along heading conditions, yielding close to zero path error. The opposite occurs when the path radius increases—the peak in the gaze along heading condition (blue *) shifts leftward in Figure 7b, yielding larger path error.

### Path Perception and Eye Movements

**Figure 8:**
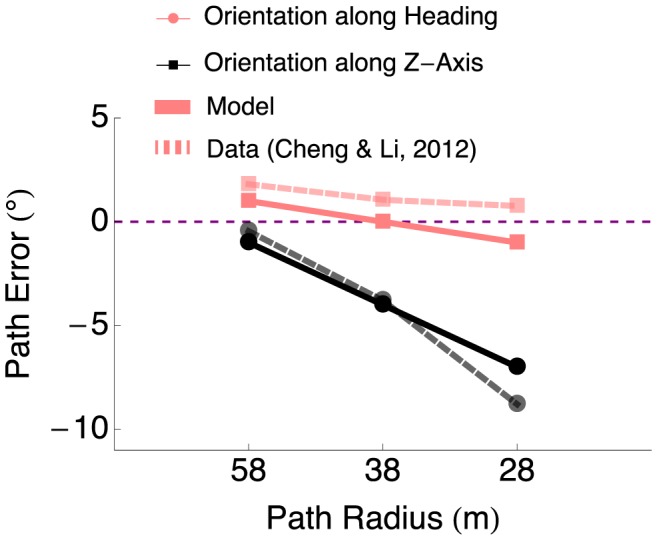
Model path error in conditions that involve smooth pursuit eye movements. The optic flow that appears on the observer retinal during smooth pursuit of a horizontally moving target is identical in the orientation along heading and orientation along Z-axis conditions. The orientation along heading condition is similar to the gaze along heading condition, except the observer tracks a target that moves in the direction *opposite* of the path curvature. The orientation along Z-axis condition is similar to the Z-axis condition, except the observer tracks a target that moves in the *same* direction of the path curvature. Similar to human subjects, the model yields low path errors for all the path radius conditions because model gain fields compensate in the direction opposite that of the eye movements. The model increasingly underestimates path curvature in the orientation along Z-axis condition, similar to humans.

Figure 8 plots the results of model simulations of the two experimental conditions of Cheng and Li, in which human subjects performed smooth pursuit eye movements to track a moving target [25]. Path errors produced by our model fit the human data well in both the orientation along heading (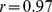) and orientation along Z axis (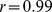) conditions. Model gain fields modulate the optic flow signal proportional to the mean eye tracking speeds of human subjects, which increase with path curvature. Model gain fields modulate the optic flow signal proportional to the mean eye tracking speeds of human subjects, which increase with path curvature. Subjects tracked a target moving in the direction of the drift in FoE position in the orientation along Z-axis condition. The only source of rotation in the optic flow field is that due to pursuit eye-movements, the gain field adds rotation in the opposite direction with a pursuit-speed proportional magnitude, effectively nulling the rotation and producing a translation-only optic flow field. The model increasingly underestimates the path curvature as the curvature increases because the combined effect of rotation due to path curvature, rotation due to eye-movements, and rotation added by the gain-fields is a rotation component of the flow field that is less than would be observed due to path curvature alone. As a result, model path errors are small.

### Heading

**Figure 9:**
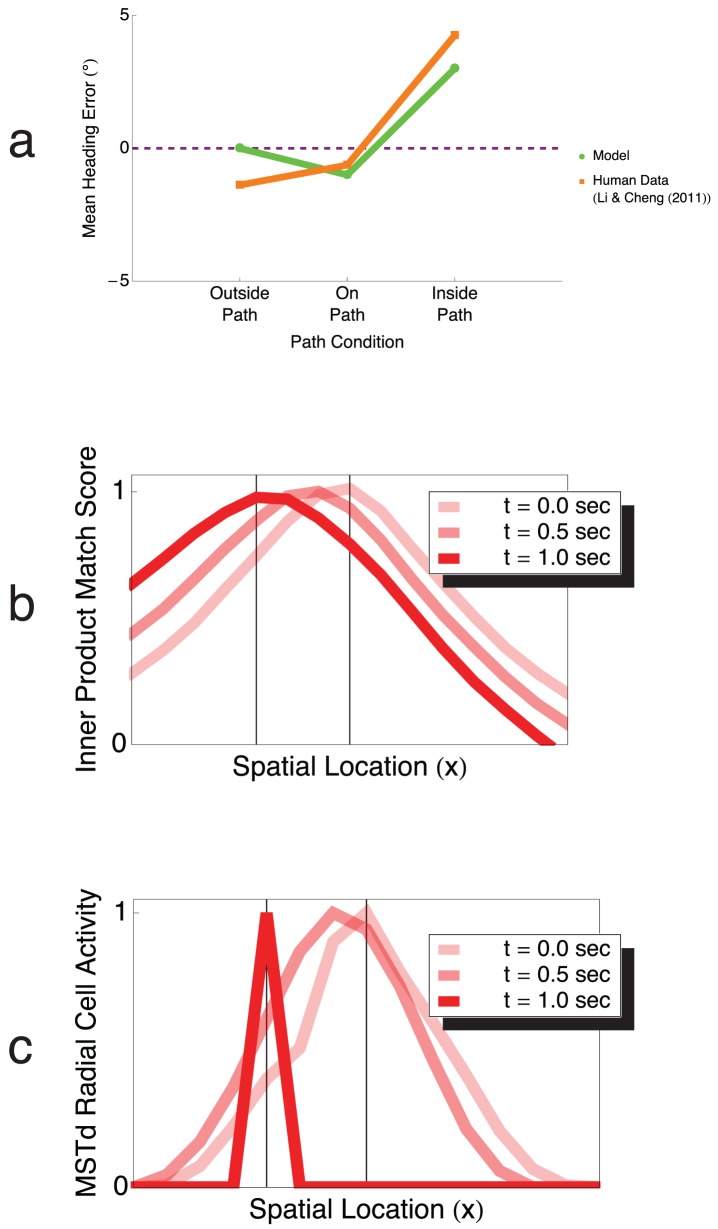
Heading errors produced by the model during travel along a circular path. Positive and negative heading errors indicate bias in heading judgments in the direction of and the direction opposite to the path curvature, respectively. The model and humans produced small negative heading errors in the on path condition, and more substantial positive bias in the inside path condition. The model yielded veridical heading performance in the outside path condition, which occurred because the model is not sensitive enough to differences in the optic flow in the outside path and Z-axis conditions. Heading bias in the model is preserved over time without (b) and with (c) competition in MSTd.

In Figure 9a, model heading bias in the outside path, on path, and inside path conditions is compared to that of human subjects in the experiments of Li and Cheng [31]. Heading is represented in the model as the preferred 2D visuotopic position of the maximally active MSTd neurons (see Materials and Methods). Positive and negative heading errors correspond to heading judgments biased in the direction of and opposite to the path curvature, respectively. Human heading judgments were slightly biased outside the path in the outside path and on path conditions, and more greatly biased toward the inside of the path in the inside path condition (Figure 9a, red) [31]. The model produced similar heading errors, but unlike the human data, model heading estimates were veridical in the outside path condition. This occurred because the model was not sensitive enough to detect differences between the MSTd activity peaks in the Z-axis and outside path conditions (Figure 7b), so the model signals the veridical heading. Neither heading errors produced by the human subjects nor by the model were influenced by the path radius.

Figures 9b–c depict the temporal evolution of the spatial distribution in MSTd among units sensitive to radial expansion without competition (Figures 9b) and with competition (Figures 9c). The x-axis corresponds to MSTd unit FoE selectivity to particular horizontal locations within the visual field. The simulation is of the Z-axis condition, wherein the instantaneous optic flow is always expanding radially without rotation, and Figures 9c shows the activity of model neurons tuned to radial expansion. The visuotopic positions of the activity peaks in MSTd do not change due to the competition, but the model competitive interactions sharpen the spatial distribution. Any heading bias therefore is preserved in the model through the competition in MSTd.

### Simulated Rotation

**Figure 10:**
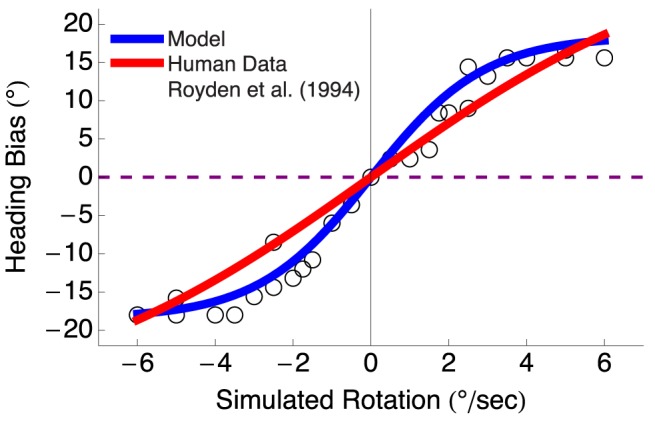
Heading bias yielded by the model in the simulated rotation condition with rotation rates between 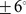. When human subjects fixate on optic flow displays wherein an observer moves along a straight path with rotation, humans make large heading errors in the direction of the simulated rotation and report the perception of travel along a curved path. The model (blue) produced the same sigmoidal pattern of heading bias as human subjects (red). Both sets of data points were fit well with a hyperbolic tangent function. The similarity between model and human heading bias, suggests the model mechanisms can explain the curved path percept reported by human subjects.

In human psychophysical studies that employ a simulated eye rotation condition, the observer moves on a straight path with an added amount of rotation [11]. However, human subjects report the perception of moving along a curved path [15]. We tested whether our model produces similar heading bias to human subjects in the simulated rotation condition, which would offer an mechanistic explanation of the curved path percepts. To compute heading bias, we compared the heading garnered by the model in the gaze along heading condition with that obtained when simulating observer travel along a straight path with added rotation rates between 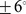. We simulated travel toward two fronto-parallel planes and otherwise mimicked experiment 2 of Royden et al. [11]. Figure 10 depicts model heading bias (blue) for different amounts of simulated rotation fitted by a hyperbolic tangent function (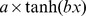, where 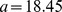 and 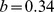, 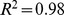). The red curve in Figure 10 shows the hyperbolic tangent function fit (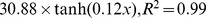) to mean human data from [11]. The sigmoidal functions fit the human data and model well, and the two were well correlated with one another (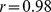). Figure 10 shows that heading was biased in the direction of the simulated rotation, which is the same sign of error observed in Figure 9a. Therefore, both the model and humans data exhibit heading bias in the simulated rotation condition, which may explain the curved path percepts in humans.

### Is Competition in MSTd Necessary?

**Figure 11:**
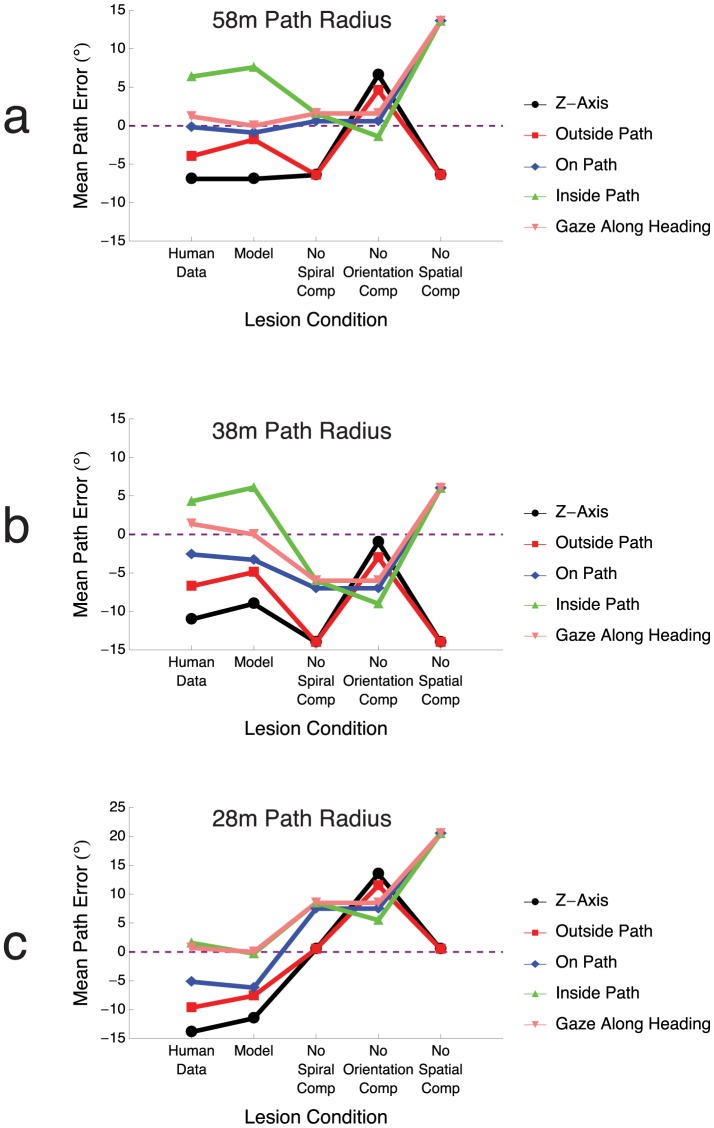
The impact lesions to model MSTd have on path error. The mean path errors for human subjects and the model from Figure 7 are plotted on the two leftmost data columns for 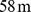 (a), 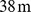 (b), 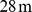 (c) radius paths. Lesions were introduced in the model MSTd connectivity by zeroing out competitive interactions in spiral space, across spiral orientation, and across 2D space between neurons in MSTd (see Eq. 6). Lesions had a detrimental impact on model performance, and path curvature estimates no longer mapped onto human judgments. The three competitive interactions in model MSTd were necessary to obtain our results.

Our model incorporates competitive dynamics across a spiral space. To determine whether competition across spirality, spiral orientation (CW v.s. CCW), and visuotopic space in model MSTd was necessary to produce path errors comparable to humans, we selectively lesioned certain competitive interactions between model neurons. Path errors were computed by comparing the peak activity in spiral space to that obtained in the gaze along heading condition, as in the unlesioned case. Figure 11 compares human and intact model mean path errors with those produced when the three types of competition in the model were lesioned. In all cases, omitting a particular type of competitive interaction in the model resulted in changes in path errors. For instance, lesioning the horizontal spatial interactions between model MSTd neurons resulted in a shift and compression in path error across all path radii: the path errors for the inside path, on path, and gaze along heading conditions converged to the same value for each path radius, and path errors in the Z-axis and outside path conditions converged to a different value. Introducing lesions into model MSTd connections garnered results that did not exhibit the same pattern as human judgments. Human behavioral performance is compatible with the use of competitive interactions between subpopulations of cells in MSTd.

### Different Dot Densities

**Figure 12:**
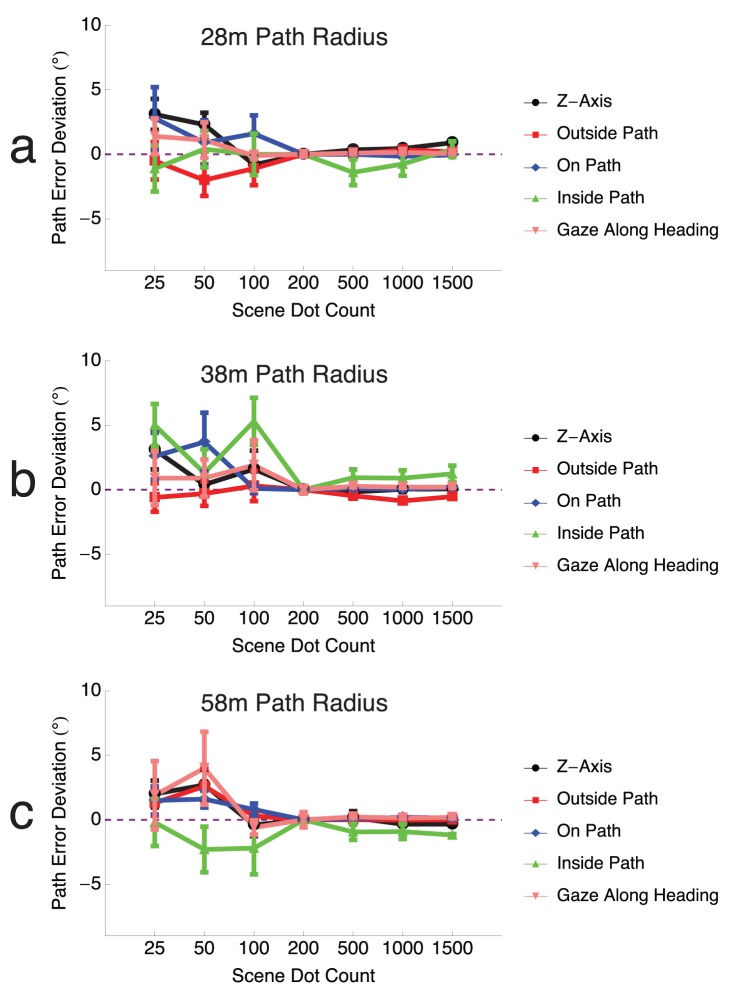
Robustness in model path curvature estimates for scenes containing 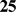
–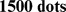. The deviation in path errors from those shown in Figure 7 are plotted for path radii of 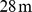 (a), 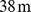 (b), and 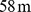 (c), respectively. Deviations in path error were modest, with mean errors falling within 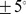 of those depicted in Figure 7. There were only small deviations in any condition when the scene contained at least 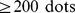.

We tested the model stability and path curvature estimation performance as a function of the number of dots in the scene. The path curvature judgments made by human subjects in the experiments of Li and Cheng [31] and the model results shown in Figure 7 were derived from environments with 200 dots. Figure 12 shows model performance across the path curvature conditions as function of scene dot count. The y axis plots the *path error deviation*, which indicates the relative path error compared to that obtained with 200 dots. Independent of the path radius, the model yields reliable results, with modest mean path error deviations (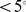) even with only 25 dots. Human path curvature judgments have been tested in environments containing different dot densities in conditions that most closely resemble those in the gaze along heading condition, and model produces similar errors to these human data [27]. Path errors in scenes with greater numbers of dots than 200 also yielded low magnitude path error deviations, which indicates that the model results shown in Figure 7 are stable and model parameters did not overfit the human data.

### Path Selective Neurons

**Figure 13:**
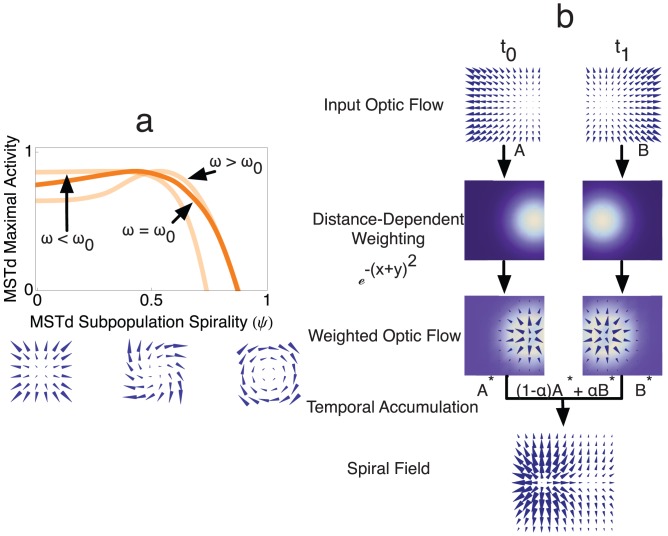
Model simulation of the experiment of Froehler and Duffy [43]. (a) Responses of the maximally-active model MSTd subpopulations in spiral space as a function of the angular rotation rate (i.e. how fast the circular path is traversed per unit of time). When the angular rotation rate matched that used by Froehler and Duffy (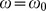, dark orange), model MSTd neurons most sensitive to spiral patterns were most active. This also occurred when for larger angular rotation rates (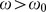). When the angular rotation was set to a comparable rate to that used in the *Z-axis* condition [31] (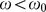), model MSTd neurons most sensitive to radial expansion elicited the maximal activation. (b) Simplified model mechanisms that explain why neurons that are sensitive to spirals produced the peak activity in spiral space in the simulation of the Froehler and Duffy experiment, but did not in the simulation of the *Z-axis* condition. Consider the first-order optic flow (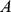 and 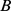) at two times (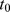 and 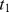) during the circular path traversal (top row). Template matching in the model is inversely weighted by distance to the FoE or CoM (second row). The third row shows the optimal templates inversely weighted by distance (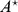 and 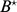). Because model MSTd dynamically integrates afferent signals from model MT, activation due to the input at 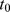 influences the activation due to the input at 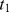. Temporal accumulation in the model can be approximated by considering 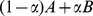, which temporally blends the two weighted fields. This yields a spiral field (bottom row), and explains why model MSTd neurons sensitive to spiral patterns are most active when the angular rotation rate about the circular path is sufficiently large.

Figure 13a shows a model simulation of first-order optic flow experienced by the monkey in the experiments of Froehler and Duffy [43]. The gaze of the monkey traveling along the circular track was tantamount to that of the Z-axis condition. Therefore, according to traditional theory, and the assumptions of the experimenters, the radial subpopulation of cells in MSTd was expected to be maximally active due to the absence of rotation in the instantaneous optic flow field. However, in our simulations the maximally active model MSTd subpopulation was tuned to spiral patterns rather than those that are radial (dark orange). When the angular rotation rate 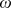 exceeded that used by Froehler and Duffy [43] (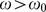), MSTd neurons in the model tuned to spiral patterns remained the most active. When the angular rotation rate was comparable to that used in the *Z-axis* condition of Li and Cheng (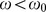), the model neurons selective to radial patterns were most active. Our analysis indicates that temporal accumulation due to the dynamical properties of the MSTd model (Eq. 6) and the distance-dependent weighting (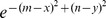, see Eq. 5) induced a peak shift in spiral space, from neurons sensitive to radial patterns to those sensitive to spirals. As shown in Figure 13b, the temporal accumulation and spatial weightings transform the sequence of radial patterns with a ‘drifting’ FoE into a spiral pattern with a fixed FoE. When the speed around the circle is slower than that of the monkey in the experiments of Froehler and Duffy, the activity in MSTd spiral space is distributed so that the subpopulation of units tuned to radial expansion is most active. At higher speeds around the circle, the position of the MSTd peak shifts so that units sensitive to spiral patterns are most active (Figure 13). The peak shift occurs at higher speeds because the temporal dynamics ‘blur’ the flow fields and the spatial weighting distorts the flow near the FoE. Our analysis suggests that the path selective neurons identified by Froehler and Duffy in MSTd are in fact preferentially tuned to spiral patterns, and the spiral space competition employed in our model can explain the mechanism underlying their path selective properties. We predict that if Froehler and Duffy performed their experiment at slower rotation rates, decreased spatio-temporal accumulation would occur and fewer MSTd neurons would yield a differential CCW versus CW path selectivity. In addition, we predict that if Li and Cheng simulate travel along circular paths at faster rotation rates in the Z-axis condition, subjects would underestimate path curvature to a lesser degree.

## Discussion

In this article, we present experiments using a computational model of the primate dorsal stream to test our claim that area MSTd can simultaneously code heading and path curvature. We posit that the underlying mechanism involves competition between neurons in MSTd that are sensitive to large field spiral motion patterns. Electrophysiological data support our definition of MSTd spiral tuning space as a continuum, ranging from radial expansion or contraction to CW and CCW center motion patterns (Figure 3). We tested our spiral coding hypothesis through model simulations of observers moving along curvilinear paths, and comparing results to those garnered by studies of human path perception. We simulated the experiments of Li and Cheng, wherein observers viewed displays simulating travel along circular paths with different radii and loci of gaze [31]. The model produced similar errors to humans that maintained five different patterns of gaze (Figure 7). This indicates that, similar to human subjects, perception of path curvature is underestimated when gaze is along a fixed direction in the world (along the ‘Z-axis’), outside the path, and on a location down the future path; overestimated with gaze is inside the path; and relatively accurate when gaze changes such that it is always in the heading direction (i.e. tangent to the circle). Figure 7b shows that the model explains the human path errors through the rank ordering of activity peaks distributed along the MSTd spiral space sensitivity continuum. Overestimations and underestimations of path curvature occur in the model because each pattern of gaze influenced the retinal rotation differently over time. This shifted activity peaks in MSTd spiral space compared to the peak yielded when the observer was simulated to look where he was going, which commonly occurs during natural locomotion. The steady-state shifts in MSTd population activity compared to the natural gaze along heading gaze condition to which the model is calibrated results in systematic biases in path curvature estimation, similar to human subjects.

The reasons for comparing MSTd activity peaks in spiral space to that obtained in the gaze along heading condition are twofold. First, directing gaze in the direction of the heading naturally occurs in many activities, such as locomotion and driving. Aligning gaze along heading appears to be important for human perception of path because only in this gaze condition did humans accurately assess the path curvature [31]. The results of Li and Cheng are supported by a number of similar studies [23], [24], [27], [28]. When human mothers carry their infants, statistics during locomotion indicate that gaze is most often maintained within 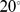 of the heading direction [60]. Second, human perception of metric space has been demonstrated to be inaccurate and it therefore would seem more likely that humans perceive path relative to conditions afforded during normal locomotion (i.e. when gaze naturally changes with heading direction) rather than perceiving path in absolute terms. For example, humans exhibit distorted judgments of distance and slant [61], [62]. The rank order of the model MSTd activity peak positions in spiral space followed that of path errors made by human subjects (Figure 7) across different gaze and path curvature conditions. This supports the idea that humans perceive their path of travel by using the pattern of MSTd activity yielded during natural location as a reference for when gaze changes.

### Path Perception in Different Visual Scenes

In the experiments of Li and Cheng, human path errors were not modulated by the structure of the visual scene [31]. Our simulations demonstrated that model performance was only modestly impacted by the dot density of the ground plane (Figure 12). This is consistent with the findings of Li and Cheng that denser textured environments did not modulate human path judgments. The robustness of the model results to dot density is also consistent with findings that indicate that path perception does not depend on local features in the environment [31]. The stability of path errors across different types of scenes in humans and the model suggests that mechanisms underlying path perception depend on areas such as MSTd that prefer stimulation by large field pattern motion.

Existing studies of path perception have explored path perception during travel along ground planes [24], [31]. We are unaware of investigations that explored other environmental structures, such as 3D dot clouds and fronto-parallel planes. Model simulations mainly contained ground plane environments due to their natural relevance to human locomotion and the availability of psychophysical data. To investigate the simulated rotation condition with our model, we mimicked the conditions of experiment 2 of Royden et al., which contained two dot fronto-parallel planes [11]. More work needs to be done to assess human path perception in different types of environments.

### Simulated Rotation

The simulated rotation condition of Royden presents an interesting test for the model [11]. We hypothesize that humans perceive that they are traveling along a curved path in the simulated rotation condition due to the activation of spiral-selective neurons in MSTd. Our hypothesis is supported by simulations that demonstrate that spiral-selective units, not those tuned to radial expansion, are maximally active in the simulated rotation condition. Conversely, in the Z-axis condition of Li and Cheng, human subjects responded as if they were traversing a straight path despite actually traveling along a curved path. In this case, model neurons tuned to radial expansion produced the most activity, which signals a lack of path curvature and is consistent with human path errors. In the simulated rotation and Z-axis conditions, the spiral space mechanisms in the model correctly predict the perceived path curvature. This suggests that humans rely on retinal rotation (i.e. rotation not due to extra-retinal sources) to perceive the curvilinear path and that MSTd neuronal tuning to spirals extracts information about path curvature. The large heading biases produced by humans in the presence of retinal rotation [11] is consistent with the finding of Orban and colleagues that MSTd neurons tuned to expansion do not appear to compensate for rotational components in the optic flow field, except when accompanied by an extra-retinal signal [44].

Interestingly, the environments and rotation rates tested by Li and Cheng and Royden et al. are remarkably similar, yet our model yields different heading bias in each of these experimental conditions reflecting the differences found in humans (compare Figure 9a and 10). In particular, experiments 4 and 5 in the Royden et al. study both use ground planes defined by random dot patterns, with rotation rates in the range of 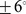. Despite these similarities in the instantaneous optic flow fields, human heading bias reached 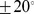 in the Royden et al. study and it did not exceed 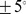 in the experiment of Li and Cheng. The difference in heading bias may be attributed to spatio-temporal differences in the optic flow displays. As a dynamical system, our model responds differently to different spatio-temporal optic flow evolution [15]. Rather than using a ground plane with a uniform dot density similar to that of Royden and colleagues, Li and Cheng distributed dots to maintain a constant density at different depths within the observer's field of view. This manipulation increased motion parallax in the displays, which has been shown to improve the accuracy of heading judgments [36]. There were only 220 dots visible at the trial outset in the experiments of Royden and colleagues compared to 300 in the displays of Li and Cheng. Differences in motion parallax and dot density may account for the disparity in heading bias between the two studies.

It is also possible that subjects in the experiments of Royden et al. reported perceived path rather than heading. Our model provides a good quantitative fit to the heading bias in both studies. Only the spatio-temporal structure of the displays used to simulate the studies differed. If we assume that human subjects followed the experimental instructions, then our fit of the data is consistent with the reporting of heading. However, if we assume that subjects attempted to indicate the curvature of their path, then the interpretation of our data fit is incorrect.

### Representation of Path Curvature in MSTd

The activity curves in model MSTd spiral space (Figure 7*b*) exhibit different widths and sharpnesses. Because model MSTd was configured as a soft winner-take-all network (Eq. 6), given sufficient time, the network will select a single MSTd unit to be active and all other model neurons will be suppressed through competition. The winning unit signals the path curvature through its pattern selectivity in spiral space. As noted in other computational studies [51]–[53], [57], broad activation in the network could implicate a greater degree of uncertainty about the path curvature and the dynamical competitive interactions require longer to resolve a high confidence solution. We configured model MSTd with a single set of parameters, but it is possible *in vivo* that different subpopulations exhibit differential response latencies [63].

As depicted in Figure 7b, simulations of travel along curved paths gives rise to complex distributions of activity across MSTd that are important to how the model encodes heading and path. It is unclear how the brain decodes this information that is distributed across the MSTd population. We believe the population activity is important to heading and path perception, and taking the argmax just provides a simple and straightforward method to assess model performance and properties about the MSTd population. Because competitive dynamics occur while input optic flow signals remain present, a total suppression of the activity of the non-winning units is not guaranteed to occur [51], [63]. The argmax operation allows us to read out information about the most active model unit to understand model performance, and is not part of the model's operation. The winner-take-all mechanism is part to the model's operation, and as indicated by our results (Figure 11), represents an important characteristic of the model that allows it to fit the human data.

We selected spiral templates in the model to resemble the optic flow patterns used in a number of electrophysiological studies [45], [47], [64] to investigate large motion pattern selectivity in neurons located in MSTd and other areas of the STS. Although electrophysiological studies report tuning in the spiral space that spans radial expansion, contraction, and center fields, actual MSTd neuron receptive fields may exhibit far greater complexity. Pack and colleagues modeled the feedforward subunit structure of MSTd neurons based on single-cell recordings and discovered complicated subunit configurations that deviated from characteristic radial, spiral, and center motion patterns [49]. Feedback and other types of horizontal connectivity was not modeled, and only 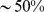 of the MSTd response variance was accounted for, so the actual receptive fields of MSTd units are likely even more complex. MSTd receptive fields may follow the motion statistics experienced by primates during ecological locomotion along a ground surface. For instance, model templates spanned the entire visual field, but ‘ecological templates’ may be biased toward the lower portion of the visual field. The statistics of videos collected from head-mounted cameras on human mothers carrying infants show that the optic flow during locomotion is fairly evenly distributed across expanding, contracting, upward, downward, CW, and CCW motion patterns, with a bias for expansion [60]. The selectivity of MSTd neurons in the sample of Graziano and colleagues also are biased toward expansive motion patterns. Humans accurately judge heading in environments with many different structures, even with dynamic occlusion, unless the textures become unstructured [30]. Therefore, ecological statistics may be important for guiding the development of MSTd receptive fields.

### Path Selective Cells

In simulating monkey movement along a circular path, we found that the location of the MSTd activity peak in spiral space depended on the speed at which the circular path is traversed. At speeds slower around the circular track than that used by Froehler and Duffy, the optic flow more closely mimicked the Z-axis condition of Li and Cheng [31], and the subpopulation of MSTd neurons tuned to radial expansion was most active—thereby signaling travel along a straight path. However, when the path traversal speed equaled or exceeded that used in the study of Froehler and Duffy [43], the activity peak shifted rightward, signaling navigation along a curved path. Our analysis indicates that at a sufficiently fast speed around the track, the motion signal MSTd neurons receive in the experiment of Froehler and Duffy is temporally ‘blurred’ and actually resembles a spiral pattern (Figure 13). Froehler and Duffy did not report testing selectivity to spirals in their sample. Our analysis and simulation results predict that the path selective neurons discovered by Froehler and Duffy were tuned to spirals rather than expansion patterns. We predict that human subjects would produce path curvature judgments consistent with the percept of traveling along a curved path in a psychophysical experiment with the Z-axis gaze condition when the rotation rate along the circle is increased. In this proposed experiment, the model makes the prediction that humans would produce different path errors in the *Z-axis condition*, depending on how much of and the speed at which the circular path is traversed.

Our model results suggest information about future path may be processed in areas as early as MSTd. Path estimation may more fundamentally indicate the functional role of area MSTd in primates.

## References

[1] Gibson JJ (1979) The Ecological Approach To Visual Perception. Psychology Press : 1–174.

[2] Warren WH (1998) The State of Flow. In: Watanabe T, editor, High-level motion processing, Cambridge: MIT Press. pp. 315–358.

[3] Warren WHW, Morris MWM, Kalish MM (1988) Perception of translational heading from optical flow. Journal of experimental psychology Human perception and performance 14: 646–660. 2974874 10.1037//0096-1523.14.4.646

[4] van den Berg AVA (1992) Robustness of perception of heading from optic flow. Vision research 32: 1285–1296. 1455703 10.1016/0042-6989(92)90223-6

[5] van den Berg AV (1996) Judgements of heading. Vision research 36: 2337–2350. 8776499 10.1016/0042-6989(95)00247-2

[6] Rieger JHJ, Lawton DTD (1985) Processing differential image motion. J Opt Soc Am A 2: 354–360. 3973767 10.1364/josaa.2.000354

[7] Hildreth EC (1992) Recovering heading for visually-guided navigation. Vision research 32: 1177–1192. 1509710 10.1016/0042-6989(92)90020-j

[8] Warren WH, Saunders JA (1995) Perceiving heading in the presence of moving objects. Perception 24: 315–331. 7617432 10.1068/p240315

[9] Royden CS (2002) Computing heading in the presence of moving objects: a model that uses motion-opponent operators. Vision research 42: 3043–3058. 12480074 10.1016/s0042-6989(02)00394-2

[10] Warren WHW, Hannon DJD (1990) Eye movements and optical flow. J Opt Soc Am A 7: 160–169. 2299447 10.1364/josaa.7.000160

[11] Royden CS, Crowell JA, Banks MS (1994) Estimating heading during eye movements. Vision research 34: 3197–3214. 7975351 10.1016/0042-6989(94)90084-1

[12] Royden CS, Banks MSM, Crowell JAJ (1992) The perception of heading during eye movements. Nature 360: 583–585. 1461280 10.1038/360583a0

[13] Ehrlich SM, Beck DM, Crowell JA, Freeman TC, Banks MS (1998) Depth information and perceived self-motion during simulated gaze rotations. Vision research 38: 3129–3145. 9893821 10.1016/s0042-6989(97)00427-6

[14] Li L, Sweet BT, Stone LS (2006) Humans can perceive heading without visual path information. Journal of Vision 6: 2–2. 10.1167/6.9.2 17083281

[15] Royden CS (1994) Analysis of misperceived observer motion during simulated eye rotations. Vision research 34: 3215–3222. 7975352 10.1016/0042-6989(94)90085-x

[16] Bradley DCD, Maxwell MM, Andersen RAR, Banks MSM, Shenoy KVK (1996) Mechanisms of heading perception in primate visual cortex. Science 273: 1544–1547. 8703215 10.1126/science.273.5281.1544

[17] Shenoy KVK, Bradley DCD, Andersen RAR (1999) Inuence of gaze rotation on the visual response of primate MSTd neurons. Journal of Neurophysiology 81: 2764–2786. 10368396 10.1152/jn.1999.81.6.2764

[18] Shenoy KV (2002) Pursuit Speed Compensation in Cortical Area MSTd. Journal of Neurophysiology 88: 2630–2647. 12424299 10.1152/jn.00002.2001

[19] Churchland MM, Priebe NJ, Lisberger SG (2005) Comparison of the Spatial Limits on Direction Selectivity in Visual Areas MT and V1. Journal of Neurophysiology 93: 1235–1245. 15483064 10.1152/jn.00767.2004 PMC2603170

[20] Elder DM, Grossberg S, Mingolla E (2009) A neural model of visually guided steering, obstacle avoidance, and route selection. Journal of experimental psychology Human perception and performance 35: 1501–1531. 19803653 10.1037/a0016459

[21] Rushton SK, Harris JM, Lloyd MR, Wann JP (1998) Guidance of locomotion on foot uses perceived target location rather than optic flow. Current Biology 8: 4–4. 10.1016/s0960-9822(07)00492-7 9799736

[22] Fajen BR, Warren WH (2007) Behavioral dynamics of intercepting a moving target. Experimental Brain Research 180: 303–319. 17273872 10.1007/s00221-007-0859-6

[23] Fajen BR, Kim NG (2002) Perceiving curvilinear heading in the presence of moving objects. Journal of experimental psychology Human perception and performance 28: 1100–1119. 12421058 10.1037//0096-1523.28.5.1100

[24] Saunders JA (2010) View rotation is used to perceive path curvature from optic flow. Journal of Vision 10: 25. 21149308 10.1167/10.13.25

[25] Cheng JCK, Li L (2012) Effects of reference objects and extra-retinal information about pursuit eye movements on curvilinear path perception from retinal flow. Journal of Vision 12. 10.1167/12.3.12 22410585

[26] Li L, Cheng JCK (2011) Heading but not path or the tau-equalization strategy is used in the visual control of steering toward a goal. Journal of Vision 11: 20–20. 22036919 10.1167/11.12.20

[27] Warren WH, Mestre DR, Blackwell AW, Morris MW (1991) Perception of circular heading from optical flow. Journal of experimental psychology Human perception and performance 17: 28–43. 1826318 10.1037//0096-1523.17.1.28

[28] Saunders JA, Ma KY (2011) Can observers judge future circular path relative to a target from retinal flow? Journal of Vision 11: 16–16. 21690187 10.1167/11.7.16

[29] Kim NG, Fajen BR, Turvey MT (2000) Perceiving circular heading in noncanonical flow fields. Journal of experimental psychology Human perception and performance 26: 31–56. 10696604 10.1037//0096-1523.26.1.31

[30] Kim NG (2008) Dynamic Occlusion and Optical Flow From Corrugated Surfaces. Ecological Psychology 20: 209–239.

[31] Li L, Cheng JCK (2011) Perceiving path from optic flow. Journal of Vision 11: 22–22. 21270114 10.1167/11.1.22

[32] Stone LS, Perrone JA (1997) Human heading estimation during visually simulated curvilinear motion. Vision research 37: 573–590. 9156201 10.1016/s0042-6989(96)00204-0

[33] Li L, Warren WH Jr (2004) Path perception during rotation: inuence of instructions, depth range, and dot density. Vision research 44: 1879–1889. 15145682 10.1016/j.visres.2004.03.008

[34] Li L, Chen J, Peng X (2009) Inuence of visual path information on human heading perception during rotation. Journal of Vision 9: 29–29. 19757968 10.1167/9.3.29

[35] Lee DN, Lishman R (1977) Visual control of locomotion. Scandinavian Journal of Psychology 18: 224–230. 897600 10.1111/j.1467-9450.1977.tb00281.x

[36] Li L, Warren WH (2000) Perception of heading during rotation: sufficiency of dense motion parallax and reference objects. Vision research 40: 3873–3894. 11090678 10.1016/s0042-6989(00)00196-6

[37] Wann JP, Swapp DK (2000) Why you should look where you are going. Nature neuroscience 3: 647–648. 10862695 10.1038/76602

[38] Land MF, Lee DN (1994) Where we look when we steer. Nature 369: 742–744. 8008066 10.1038/369742a0

[39] Orban GA (2008) Higher Order Visual Processing in Macaque Extrastriate Cortex. Physiological Reviews 88: 59–89. 18195083 10.1152/physrev.00008.2007

[40] Duffy CJ, Wurtz R (1997) Medial superior temporal area neurons respond to speed patterns in optic flow. The Journal of neuroscience 17: 2839–2851. 9092605 10.1523/JNEUROSCI.17-08-02839.1997 PMC6573103

[41] Duffy CJ, Wurtz R (1995) Response of monkey MST neurons to optic ow stimuli with shifted centers of motion. The Journal of neuroscience 15: 5192–5208. 7623145 10.1523/JNEUROSCI.15-07-05192.1995 PMC6577859

[42] Britten KH (2008) Mechanisms of Self-Motion Perception. Annual Review of Neuroscience 31: 389–410. 10.1146/annurev.neuro.29.051605.112953 18558861

[43] Froehler MT, Duffy CJ (2002) Cortical neurons encoding path and place: where you go is where you are. Science 295: 2462–2465. 11923540 10.1126/science.1067426

[44] Orban GAG, Lagae LL, Verri AA, Raiguel SS, Xiao DD, et al (1992) First-order analysis of optical flow in monkey brain. PNAS 89: 2595–2599. 1557363 10.1073/pnas.89.7.2595 PMC48708

[45] Graziano MSM, Andersen RAR, Snowden RJR (1994) Tuning of MST neurons to spiral motions. The Journal of neuroscience 14: 54–67. 8283251 10.1523/JNEUROSCI.14-01-00054.1994 PMC6576843

[46] Schaafsma SJS, Duysens JJ (1996) Neurons in the ventral intraparietal area of awake macaque monkey closely resemble neurons in the dorsal part of the medial superior temporal area in their responses to optic ow patterns. Journal of Neurophysiology 76: 4056–4068. 8985900 10.1152/jn.1996.76.6.4056

[47] Read HLH, Siegel RM (1997) Modulation of responses to optic flow in area 7a by retinotopic and oculomotor cues in monkey. Cerebral cortex (New York, NY : 1991) 7: 647–661. 10.1093/cercor/7.7.647 9373020

[48] Born R, Bradley D (2005) Structure and function of visual area MT. Annual Review of Neuroscience 28: 157–189. 10.1146/annurev.neuro.26.041002.131052 16022593

[49] Mineault PJ, Khawaja FA, Butts DA, Pack CC (2012) Hierarchical processing of complex motion along the primate dorsal visual pathway. Proceedings of the National Academy of Sciences of the United States of America 109: E972–80. 22308392 10.1073/pnas.1115685109 PMC3341052

[50] Grossberg S (1973) Contour enhancement, short term memory, and constancies in reverberating neural networks. Studies in applied Mathematics 52: 213–257.

[51] Layton OW, Mingolla E, Browning NA (2012) A motion pooling model of visually guided navigation explains human behavior in the presence of independently moving objects. Journal of Vision 12: 1–20. 10.1167/12.1.20 22275469

[52] Browning NA, Grossberg S, Mingolla E (2009) A neural model of how the brain computes heading from optic flow in realistic scenes. Cognitive psychology 59: 320–356. 19716125 10.1016/j.cogpsych.2009.07.002

[53] Browning NA, Grossberg S, Mingolla E (2009) Cortical dynamics of navigation and steering in natural scenes: Motion-based object segmentation, heading, and obstacle avoidance. Neural Networks 22: 1383–1398. 19502003 10.1016/j.neunet.2009.05.007

[54] Raudies F, Neumann H (2012) A review and evaluation of methods estimating ego-motion. Computer Vision and Image Understanding 116: 606–633.

[55] Longuet-Higgins HC, Prazdny K (1980) The interpretation of a moving retinal image. Proceedings of the Royal Society of London Series B: Biological Sciences 208: 385. 10.1098/rspb.1980.0057 6106198

[56] Grossberg SS, Mingolla EE, Pack CC (1999) A neural model of motion processing and visual navigation by cortical area MST. Cerebral cortex (New York, NY : 1991) 9: 878–895. 10.1093/cercor/9.8.878 10601006

[57] Browning NA (2012) A Neural Circuit for Robust Time-to-Contact Estimation Based on Primate MST. Neural Computation 24: 2946–63. 22845825 10.1162/NECO_a_00347

[58] Layton OW, Browning NA (2013) The Simultaneous Coding of Heading and Path in Primate MSTd. In: The 2013 International Joint Conference on Neural Networks (IJCNN); 4–9 August 2013; Dallas, Texas, United States of America. Available: http://www.ijcnn2013.org/

[59] Kim NG, Turvey MT (1999) Eye Movements and a Rule for Perceiving Direction of Heading. Ecological Psychology 11: 233–248.

[60] Raudies F, Gilmore RO, Kretch KS, Franchak JM, Adolph KE (2012) Understanding the Development of Motion Processing by Characterizing Optic Flow Experienced by Infants and their Mothers. Proceedings of ICDL-EpiRob 2012: 1–6.

[61] Norman JF, Todd JT, Perotti VJ, Tittle JS (1996) The visual perception of three-dimensional length. Journal of experimental psychology Human perception and performance 1: 173–186. 8742260 10.1037//0096-1523.22.1.173

[62] Witt JK, Proffitt DR, Epstein W (2004) Perceiving distance: A role of effort and intent. Perception 33: 577–590. 15250663 10.1068/p5090

[63] Layton OW, Browning NA (2012) Recurrent competition explains temporal effects of attention in MSTd. Frontiers in Computational Neuroscience 6: 1–13. 23060788 10.3389/fncom.2012.00080 PMC3464456

[64] Duffy CJ, Wurtz R (1991) Sensitivity of MST neurons to optic flow stimuli. I. A continuum of response selectivity to large-field stimuli. Journal of Neurophysiology 65: 1329–1345. 1875243 10.1152/jn.1991.65.6.1329

